# Lucid Dreaming: Intensity, But Not Frequency, Is Inversely Related to Psychopathology

**DOI:** 10.3389/fpsyg.2018.00384

**Published:** 2018-03-22

**Authors:** Liat Aviram, Nirit Soffer-Dudek

**Affiliations:** The Consciousness and Psychopathology Laboratory, Department of Psychology, Ben-Gurion University of the Negev, Beer-Sheva, Israel

**Keywords:** lucid dreams, control, induction, emotional valence, psychopathology, depression, anxiety, stress

## Abstract

Lucid dreaming (LD) is awareness that one is dreaming, during the dream state. However, some define and assess LD relying also on controlling dream events, although control is present only in a subset of lucid dreams. LD has been claimed to represent well-being, and has even been used as a therapeutic agent. Conversely, LD is associated with mixed sleep-wake states, which are related to bizarre cognitions, stress, and psychopathology, and have been construed as arousal permeating and disrupting sleep. We propose that previous conflicting findings regarding relations between LD and both psychopathology and well-being, stem from the non-differentiated assessment of frequency and control. The present study aimed to develop an expansive measure of several LD characteristics (the Frequency and Intensity Lucid Dream questionnaire; FILD), and explore their relations with symptomatology. Undergraduate students (*N* = 187) self-reported trait LD, psychopathology (depression, anxiety, obsessive-compulsive symptoms, dissociation, and schizotypy), stress, and sleep problems; 2 months later, a subsample (*n* = 78) reported psychopathology again, and also completed a dream diary each morning for 14 days. Preliminary evidence supports the reliability and validity of the FILD. Items converged into four domains: frequency, intensity (e.g., control, activity, certainty of dreaming), emotional valence, and the use of induction techniques. We report an optimal frequency cutoff score to identify those likely to experience LD within a 2-week period. Whereas LD frequency was unrelated to psychopathology, LD intensity, and positive LD emotions, were inversely associated with several psychopathological symptoms. Use of deliberate induction techniques was positively associated with psychopathology and sleep problems. Additionally, we demonstrated directionality by employing a prospective-longitudinal design, showing that deliberate LD induction predicted an increase in dissociation and schizotypy symptoms across 2 months. We conclude that lucidity should not be considered as necessarily suggestive of well-being; LD may be positive or negative, depending on lucidity characteristics. Additionally, deliberate LD induction may harbor negative long-term risk.

## Introduction

Lucid dreaming (LD) is dreaming while being aware that one is in the dream state; La Berge et al. (1981) have empirically recorded this phenomenon by demonstrating that lucid dreamers may signal their awareness in the midst of rapid eye movement (REM) sleep. La Berge et al. (1981) characterize LD consciousness as clear and coherent, very much similar to waking cognition (see also Tholey, 1989). Although awareness of dreaming while still in the dream state is the classic and parsimonious definition of LD, many have elaborated on this definition by noting that dream awareness may lead to an ability to control, and volitionally regulate, the contents of the dream (e.g., Tart, 1988); however, the extent to which control or volition may be central or essential parts of LD is unclear. Indeed, among children and adolescents, only 37% were regularly able to change or control events in their lucid dreams (Voss et al., 2012).

The inconsistency in defining LD has led to inconsistency in LD measurement as well. Many LD studies have relied on one simply phrased question, such as “how often do you experience so-called lucid dreams?” (Schredl and Erlacher, 2004) or “have you ever had a dream during which you knew that you were dreaming?” (Alvarado and Zingrone, 2007). Assessment with a single item may overlook important aspects of LD. A slightly longer scale is the LD factor of the Iowa Sleep Experiences Survey (ISES; Watson, 2001), which includes three items, assessing both awareness and control, summed up to a single LD frequency score. Although superior to 1-item assessments, merging the 3 items to a single score may similarly overlook complexity in LD phenomenology. Conversely, some researchers have begun to look at LD as a multifaceted phenomenon. For example, a recent study differentiated between LD awareness and LD control (Harb et al., 2016). Others have addressed issues such as whether one continued to dream after achieving lucidity (Blagrove and Tucker, 1994), experienced lucidity spontaneously or by deliberate induction (Taitz, 2011), or adopted an active or passive attitude toward the dream scenario after achieving lucidity (Stumbrys et al., 2014). Voss et al. (2013) combined several such characteristics into a comprehensive LD measure, thus presenting an appropriate alternative to previous shorter measures.

The differentiation between these various aspects of LD characteristics on an empirical level is crucial for advancing the field on the theoretical level as well. For example, the occurrence of LD has been related to an internal locus of control (Blagrove and Tucker, 1994; Blagrove and Hartnell, 2000), and psychological resilience in the face of exposure to terrorism (Soffer-Dudek et al., 2011b). In fact, many researchers claim that LD is associated with mental health and well-being (e.g., Snyder and Gackenbach, 1988; LaBerge, 2014). Elevated LD frequency was associated with a better ability “to manage or control various aspects of cognitive, emotional, and social functioning while awake” (Gruber et al., 1995, p. 7) and with higher levels of mental health, assertiveness, autonomy, and self-confidence (Doll et al., 2009). However, it is likely that these characteristics pertain to those experiencing control over their dreams, whereas they may not necessarily be relevant to those experiencing dream awareness with no dream control. Indeed, studies examining the utility of LD as a therapeutic agent have shown mixed findings. Overall, research on lucid dreaming treatment (LDT) (i.e., training in lucidity induction techniques aiming to improve mental health), usually implemented on nightmare sufferers trained to alter the frightening dream scenario, has demonstrated preliminary evidence of effectiveness (Brylowski, 1990; Zadra and Pihl, 1997; Spoormaker et al., 2003; Spoormaker and van den Bout, 2006; Harb et al., 2016; see Gavie and Revonsuo, 2010 for a review). However, LDT does not seem to be superior to other treatments, such as imagery rehearsal therapy (Lancee et al., 2010), and is not effective for post-traumatic stress disorder (PTSD) (Spoormaker and van den Bout, 2006; Harb et al., 2016). Moreover, the mechanism of change following LDT is unclear, as several participants in these studies showed a reduction of symptoms without achieving lucidity (Zadra and Pihl, 1997; Spoormaker et al., 2003; Spoormaker and van den Bout, 2006). Harb et al. (2016) used ISES items separately and found that dream awareness seemed to be higher among post-traumatic veterans compared to norms from non-clinical samples, whereas dream content control was low. Moreover, they showed that the reduction in nightmare distress was related to an increase in dream content control, suggesting that the element of control, rather than dream awareness *per se*, is probably the central mechanism contributing to well-being^1^.

The lack of differentiation between LD characteristics in most of these studies means that we do not yet fully understand the correlates or consequences of *dream awareness per se* (not necessarily accompanied by volitional control). Watson (2001) construed LD, along with other unusual dream phenomena, as representing nocturnal manifestations of an “unusual cognitions” continuum (e.g., bizarreness, oddity), which during daytime is characterized by schizotypy and dissociative experiences. Notably, LD represents a “mixed state” between waking and sleeping consciousness (Mahowald and Schenck, 2001; Voss et al., 2009); other unusual dream phenomena representing mixed sleep-wake states (e.g., nightmares, recurring dreams, vivid dreams, kinesthetic dreams, and hypnagogic hallucinations) have been consistently positively associated with several psychopathological symptoms (such as depression and anxiety), stress, and negative affect, and have been viewed as representing an intrusion of waking arousal into the sleeping consciousness (Soffer-Dudek and Shahar, 2011; Soffer-Dudek, 2017a). In fact, they seem to be an early marker of covert psychological distress (Soffer-Dudek and Sadeh, 2013; Soffer-Dudek, 2016), and may be viewed as an indicator of poor sleep quality (Soffer-Dudek, 2017a). Thus, experiencing waking awareness within the dream state may be hypothesized to relate to psychopathological distress variables. Moreover, LD onset is often triggered by a nightmare (LaBerge and DeGracia, 2000; e.g., the thought “this can’t really be happening” may trigger the realization that it is not), and nightmares are strongly associated with psychopathology (e.g., Chivers and Blagrove, 1999; Roberts and Lennings, 2006). However, it is possible that some utilize this realization to exert control and alter the negative contents of the dream scenario, or to modify the bad dream into a pleasant one. Indeed, in one study, 65% of respondents reported their LD to have a positive impact on them (Wolpin et al., 1992). It has been suggested that experiencing arousal in sleep as an intrusion, as opposed to experiencing it along with control and volition, may explain why LD does not show a consistent relation with psychopathology as do the other mixed states (Soffer-Dudek, 2017a).

Indeed, when examining the associations of LD with distress variables, inconsistent results emerge: in several domains, LD was positively correlated with distress in some studies but not in others. Again, this is probably because research has not differentiated between various LD properties. For example, inconsistencies emerged regarding depressive symptoms (see Soffer-Dudek and Shahar, 2011, compared to Taitz, 2011), general measures of psychological distress (Soffer-Dudek and Shahar, 2009, 2010, 2011), PTSD (see Harb et al., 2016, compared to van Heugten–van der Kloet et al., 2014), daily life stress (Soffer-Dudek and Shahar, 2009, 2011), and dissociative symptoms (see Watson, 2001; Soffer-Dudek and Shahar, 2009, 2011; Knox and Lynn, 2014; van Heugten–van der Kloet et al., 2014). Similarly, conflicting findings have emerged regarding psychotic tendencies and LD. On one hand, LD was not associated (Knox and Lynn, 2014) or only weakly correlated (Watson, 2001) with schizotypy; and schizophrenic and manic patients did not have elevated LD (Soffer-Dudek et al., 2011a). Moreover, there may be an inverse link of LD with psychosis, as cortical areas activated during LD overlap with brain regions that are impaired in psychotic patients (Dresler et al., 2015). On the other hand, LD was found to be significantly related to parapsychological experiences such as out-of-body experiences, apparitions and extrasensory perception dreams (Alvarado and Zingrone, 2007), experiences usually associated with schizotypy; and lucid dreamers are more prone to confabulation following impaired reality monitoring (Corlett et al., 2014), a characteristic of psychosis. In this regard, it is important to mention that several techniques for deliberate LD induction (such as reality testing; Levitan and LaBerge, 1989, or the reflection technique; Tholey, 1988) require, to some extent, intentional blurring of boundaries between reality and dreaming, which perhaps may in turn impair reality monitoring or bring about dissociative symptoms, in individuals who practice such techniques. To the best of our knowledge, this possibility has never been a focus of research.

In the present study, we propose that understanding the relationships of LD with well-being versus distress should rely on the examination of different LD aspects. To address this idea, we devised a novel, extensive LD questionnaire^2^, in which we sought to divide LD into two main structures: (1) *lucidity frequency*, representing how often LD occurs (LD stringently defined as awareness of dreaming while the dream is ongoing). Within the frequency factor, we made a distinction between spontaneous LD and deliberately induced LD (overall attempts and successful attempts). We also measure frequency separately for LD which is short-lived (momentary, followed by immediate awakening) versus LD which is long-lasting (prolonged); (2) *lucidity intensity*, representing additional aspects which may characterize LD, including control over dream events, activity of the lucid dreamer, feeling certainty regarding the fact of dreaming, and perceived LD length. In addition, we also sought to assess the emotional valence characterizing LD, both *before* and *after* achieving lucidity (because emotion may change following the realization that one is dreaming).

Thus, our first aim in the current study was to develop and validate a LD measure, which we label the FILD questionnaire, addressing the manifold properties of LD, and to explore their frequencies and interrelations. We wished to validate this measure using: (a) an existing trait measure of LD, and (b) a 2-week daily diary period, where LD is reported each morning; such assessment is less biased than trait measures because less time has elapsed between the occurrence of the event and its measurement. Moreover, we wished to utilize these data to identify an optimal cutoff score for the FILD, which may predict with optimal specificity and sensitivity whether one will have a lucid dream within a 2-week assessment period. Identifying such a cutoff score may aid future daily diary research on lucid dreamers, in the initial screening of the sample. We hypothesized (*H1*) that the FILD will demonstrate adequate validity and reliability.

Our second aim was to investigate the associations of various LD characteristics with psychopathological distress, and thus clarify the possible beneficial versus detrimental aspects of LD. Possibly, dream awareness may be related to distress only when it is experienced as intrusive, rather than controllable. Similarly, associations of LD with well-being or resilience may be rooted in intensity variables, such as a sense of ability in controlling and altering dream events, or the tendency to take on an active stance or react with positive emotion following the realization that one is dreaming. In other words, the degree to which one feels control over events may linger from waking to sleeping, in accordance with the continuity hypothesis of dreaming (i.e., the idea that dreams reflect waking-life experiences, concerns, and personality characteristics; Domhoff, 2003). We hypothesized (*H2*) that lucidity characterized by high intensity (e.g., control, activity) and positive affect will relate to better sense of control not only over dreams, but also over events, behaviors, thoughts and emotions of waking life, resulting in fewer psychopathological tendencies.

Finally, because LD is a hybrid state of waking and sleeping, related to impaired reality monitoring, it may represent blurred sleep-wake boundaries and sleep disruption. As sufficient good-quality sleep is crucial for mental health (e.g., Benca et al., 1992; Kahn-Greene et al., 2007), we wished to explore whether spontaneous or deliberately induced LD will predict *change over time* in psychopathological symptoms. Thus, we used a prospective-longitudinal design, assessing psychopathology twice (with a 2-month lag). We hypothesized (*H3*) that deliberate LD induction (which usually relies on reality testing) may have a deleterious effect on sleep-wake boundaries, which may result in dissociative symptoms (see van der Kloet et al., 2012 for a conceptualization of dissociation as stemming from a labile sleep-wake cycle). Notably, we also assessed sleep problems in this study, because of the close relation of sleep to psychopathology as well as to dreaming variables.

## Materials and Methods

### Participants and Procedure

The study included two phases. Participants in the *trait phase* were 187 undergraduate students from the Department of Psychology at Ben-Gurion University of the Negev [*n* = 133 (71%) female^3^, age *M* = 23.39, *SD* = 1.45, range 18–28]. Participants (1st-year undergraduates taking the ‘Introduction to Psychology’ course) were approached via the departmental online system for psychology experiments; they were invited to participate in a study on “dreams and emotions.” In exchange for course credit, they completed online questionnaires including (in the following order): sleep quality and sleep experiences, LD, and psychopathological symptom measures, specifically, anxiety, OC symptoms, stress, depressive symptoms, schizotypy, and dissociation.

For the *daily diary phase*, the participants from the trait phase were invited via email to participate in a non-obligatory follow-up 14-day study, conducted 2 months after the trait phase, in exchange for monetary compensation (150 NIS, approximately $40). Seventy-eight participants chose to participate [*n* = 57 (73%) female, age *M* = 23.20, *SD* = 1.49, range 19–28]. We performed independent samples *t*-tests in order to make sure there were no differences between the *n* = 78 subset and the others, in all study variables, including demographics, LD, sleep and symptom measures; there were no significant differences in any of these variables. First, they completed again the online psychopathology symptom measures, in order to obtain longitudinal data on change in psychopathology. Subsequently, for 14 days, they reported (online) sleep and dreaming every morning after awakening, and psychopathological symptoms every evening before bedtime (the latter was not used in this study).

The links between dissociation and other psychopathological symptoms, based on these data as well as daily psychopathology data, are explored in Soffer-Dudek (2017b). That publication does not utilize the data on LD, used in this study. The research was approved by Ben-Gurion University’s Human Subjects Research Committee, according to the guidelines of the Declaration of Helsinki.

### Measures

#### Part I: Development and Validation of the FILD Questionnaire (*H1*)

##### Lucid dreaming: intensity and frequency

In this study, we developed the Frequency and Intensity of Lucid Dreams questionnaire (FILD), included in full in the Supplementary Material (Supplementary Section S1). The FILD first presents a brief definition of LD. The first section, pertaining to LD frequency, includes five items (momentary LD, prolonged LD, spontaneous LD, attempt to initiate LD and success in initiating LD; the success item is applicable only to respondents who reported they attempted to initiate LD at least once). The response scale includes eight categories (0 = *“never,”* 7 = *“twice a week or more”*); it was adapted from the ISES (Watson, 2001), to which we added an option right after “never,” suggesting that LD had been present in the past but not anymore. Specifically, in the preliminary process of developing the frequency response scale, talking to lucid and non-lucid dreamers led us to speculate that some individuals had experienced frequent LD during their childhood, but no longer in adulthood. Indeed, LD is quite pronounced in young children and its incidence rate drops at about age 16 years (Voss et al., 2012). Such individuals shared with us that when they are faced with response scales of existing measures, they are unsure which response they should mark (e.g., “less than once a year” or “never”? Or perhaps the frequency with which they once experienced LDs?); as none of the existing options seem to fit their experience, this may introduce noise to the data. Thus, we included the option that LD was present during childhood but not currently. We were interested in how many participants would endorse this option, i.e., whether the experience of having LD only in childhood but not in adulthood, among young adults, is indeed prevalent or negligible. It has been demonstrated that having unusual dream phenomena confined to one’s past bares different implications to well-being than simply having them or not having them presently (Brown and Donderi, 1986).^4^

The second section, which is applicable only to respondents who reported experiencing LD of any kind at least once, includes items measuring the intensity of LD. These include items asking respondents to estimate the percentage of dreams in which they experienced confidence, versus uncertainty, regarding the fact that they are dreaming; the percentage of dreams in which they take an active versus a passive stance after achieving lucidity (i.e., the extent to which the respondent is actively participating in the events of the dream versus passively observing them); and the percentage of dreams in which they are able to control and manipulate the dream content and events volitionally, versus a sense that events are out of their control. These three items are scored on a symmetrical 5-point scale [e.g., ranging from *“I’m confident in my lucidity in the vast majority of my dreams (roughly 80–100%)”* to *“I’m uncertain in my lucidity in the vast majority of my dreams (roughly 80–100%)”*]. Also, we assessed the perceived duration of lucidity in the majority of one’s lucid dreams, using two items: estimated passage of time in seconds/minutes, and estimated number of dream scenes. These items were also measured on a 5-point scale (ranging from *“Once I realize that I’m dreaming – I wake up”* to *“Usually, the lucidity lasts for 6 min or more/4 lucid dream scenes or more”*).

In addition, four items assess the emotional valence of the lucid dreams (i.e., percentage of dreams with positive or negative emotion) before and after lucidity onset. The response scale for valence has 11 points, ranging from 0% *– “no lucid dream starts as a positive dream”* to *100% – “all lucid dreams starts as a positive dream.”*

The FILD has an additional optional section, intended for research on the use of LD induction techniques. This section, if administered, should be completed only by respondents who reported that they had attempted to deliberately induce LD in the past at least once. It assesses the frequency of using seven common techniques designed to induce LD, such as keeping a dream diary and performing reality checks (see LaBerge and Rheingold, 1990 for elaboration on LD induction techniques, which aided us in composing the response scale). Assessment relies on a 5-point response scale (0 = *“never,”* 4 = *“three times a week or more during the last month”*).

Reliability (Cronbach’s alpha) for all the scales of the FILD will be reported in the results section.

##### A daily dream diary

During the daily phase of the study, each morning, participants completed a dream diary regarding their nocturnal experiences. For this purpose, we developed a daily version of the FILD (included in Supplementary Section S2). Participants are asked whether they experienced LD, and if they answer affirmatively, they are presented with additional questions exploring the characteristics of the lucidity (e.g., whether the experience was spontaneous or initiated, controlled volitionally or not, the level of tangibility of the dream, the level of confidence in dream awareness). We did not calculate reliability (Cronbach’s alpha) for the daily FILD, since each item was designed to measure a different aspect of LD on a different scale. Repeated measures reliability was also impossible to calculate because of the sparsity of LD during the daily diary phase. In the present study we use only the item asking all respondents whether they had experienced LD in the previous night or not, again due to the sparsity of LD occurrence (the specific characteristic questions apply only to those who experienced LD and thus rely on a small sample, questioning generalizability. Interested readers may request results on these items from the corresponding author).

##### The Iowa Sleep Experiences Survey (ISES; Watson, 2001)

The ISES is an additional LD measure which was administered in the trait phase of our study. The ISES measures a wide range of nocturnal experiences on a 7-point Likert scale (1 = *“never,”* 7 = *“several times a week”*), and consists of a 15-item general sleep-related experiences subscale, not used in this study, and a 3-item lucid dreams subscale (e.g., “I am aware that I am dreaming, even as I dream”). Cronbach’s alpha for the 3-item LD subscale was α = 0.82 at Time 1 and α = 0.70 at Time 2.

#### Part II: Relationships With Psychopathology and Sleep (*H2, H3*)

##### Sleep problems

Sleep problems were assessed with the Global Sleep Assessment Questionnaire (*GSAQ*; Roth et al., 2002), a reliable and valid measure used to evaluate potential sleep problems. It consists of 15 items scored on a 4-point Likert scale (1 = *“never,”* 4 = *“always”*) referring to sleep problems in the past 4 weeks. Cronbach’s alpha for the 15 items was α = 0.75 for time 1 and α = 0.59 for time 2.

##### Depression, anxiety, and stress

We used the Beck Depression Inventory (*BDI*; Beck et al., 1961) to assess depression. It includes 21 items scored on a 0–3 scale, referring to the past 2 weeks. The BDI has previously shown good reliability and validity (Beck et al., 1988b). Cronbach’s alpha for the 21 items was α = 0.92 for time 1 and α = 0.90 for time 2. We also administered the Beck Anxiety Inventory (*BAI*; Beck et al., 1988a), which consists of 21 anxiety symptoms (e.g., “hands trembling”), with respondents being asked to indicate the extent to which they were bothered by each symptom “during the past 2 weeks, including today.” Responses are scored on a 0–3 scale (0 = *“not at all,”* 3 = *“severely”*). Cronbach’s alpha for the 21 items was α = 0.91 for time 1 and α = 0.93 for time 2. We used the Maudsley Obsessive Compulsive Inventory (*MOCI*; Hodgson and Rachman, 1977) to assess OC symptoms. The MOCI is a 30-item measure with a true/false format, producing a score range of 0–30 and aiming to estimate OC symptoms such as washing, checking, slowness and being doubtful (e.g., “I spend a lot of time every day checking things over and over again”). The MOCI is considered as a valid, reliable measure, correlating with other OCD measures, and used extensively among clinical and non-clinical populations. Cronbach’s alpha for the 30 items of the MOCI was α = 0.81 at Time 1 and α = 0.80 at Time 2. Finally, we assessed stress using the Perceived Stress Scale (*PSS*, Cohen et al., 1983). The PSS measures the degree to which situations in one’s life are appraised as stressful. It correlates with other stress measures (Cohen and Williamson, 1988). In each of its 10 items, respondents are asked how often they felt a certain way during the last month, scored on a 5-point Likert scale (1 = *never*, 4 = *very often*). Cronbach’s alpha for the 10 items of the PSS was α = 0.85 at Time 1 and α = 0.82 at Time 2.

##### Dissociative experiences and psychotic tendencies

Dissociation was measured by the revised Dissociative Experiences Scale (*DES-II*; Carlson and Putnam, 1993). The DES is a widely used self-report scale requiring respondents to estimate the percentage of the time they experience 28 dissociative phenomena on an 11-point scale (0, 10, and 20%, etc.). Three sub-factors are depersonalization-derealization, dissociative amnesia, and “absorption and imaginative involvement.” The DES measures both non-clinical and clinical dissociation, and the Hebrew version has been shown to have good psychometric properties (Somer et al., 2001). Cronbach’s alpha for the 28 items of the DES was α = 0.95 at Time 1 and α = 0.90 at Time 2. We also assessed symptoms of schizotypy, with the Magical Ideation Scale (*MIS*; Eckblad and Chapman, 1983). The MIS is of common use for the assessment of a person’s proneness to schizophrenia-like experiences and thoughts (i.e., schizotypy). It consists of 30 items answered using a true/false format to measure odd, unconventional beliefs about a variety of events and experiences (e.g., “I have had the momentary feeling that someone’s place has been taken by a lookalike”). Extensive data supports the reliability and validity of this scale (Chapman et al., 1994). Cronbach’s alpha for the 30 items of the MIS was α = 0.87 at Time 1 and α = 0.85 at Time 2.^5^

### Data Analyses

First, we estimated missingness patterns using the missing values analysis function of SPSS (version 21). Little’s MCAR test for the single items of the trait questionnaires at Time 1 was non-significant [χ^2^_(10246)_ = 9895.45, ns], suggesting that data were missing completely at random. In addition, the amount of missing data was negligible; missing data were lesser than 5%, suggesting that non-response is probably ignorable and any method of dealing with it will probably yield the same results (Tabachnick and Fidell, 2007).

#### Part I: Development and Validation of the FILD Questionnaire (*H1*)

To address *H1*, we examined the distributions of the FILD items from the trait phase, and their Pearson correlation coefficients, in order to create appropriate composite subscales. Cronbach’s alpha was then calculated for each subscale, and inter-relations between the subscales were examined. We also explore the relation of the FILD subscales to an existing measure, namely, the LD scale of the ISES. In addition, we calculated a binary variable representing whether the individual experienced a lucid dream during the dream diary phase, in order to explore its biserial correlation with the trait lucidity frequency scale for additional validity support for the FILD. Finally, using this binary item, Receiver Operating Characteristic (ROC) curve analysis was conducted (e.g., Swets, 2014), in order to assess sensitivity and specificity of the FILD and to determine an optimal cutoff score for this measure, in an attempt to predict whether one will have a lucid dream within a 2-week assessment period. The ROC curve is created by plotting the sensitivity, which represents the “true positive rate,” against the value: 1 – specificity, which represents the “false positive rate,” at various threshold settings.

#### Part II: Relationships With Psychopathology and Sleep (*H2, H3*)

Correlation coefficients were computed to assess the relationship of the FILD items and subscales with psychopathology (*H2*). In addition, trait data from Time 1 and Time 2 assessment waves were analyzed using multiple linear regression analysis with a prospective-longitudinal design, in order to explore whether LD frequency may predict *change* in psychopathology symptoms over time (controlling for baseline levels of psychopathology; *H3*).

All hypotheses were tested using the criterion of 5% chance for type I error (*p* < 0.05).

## Results

### Part I: Development and Validation of the FILD Questionnaire (*H1*)

#### Distribution of FILD Items

**FIGURE 1:**
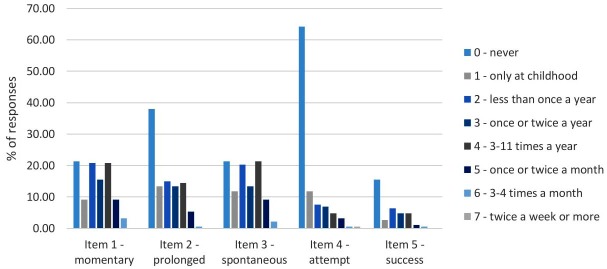
Distribution of the five frequency items of the FILD – Each shade represents a point on the frequency scale used in the FILD. FILD, Frequency and Intensity Lucid Dreaming questionnaire; Momentary, frequency of momentary LD; Prolonged, frequency of prolonged LD; Spontaneous, frequency of LD generated spontaneously; Attempt, frequency of attempts to deliberately induce LD; Success, frequency of successfully inducing LD, applicable only to those who have attempted to deliberately induce LD.

Comprehensive distribution information for the FILD items is detailed in the Supplementary Material (Supplementary Table S1). Generally, most items were at nearly normal distribution, with some exceptions; frequency of attempt to deliberately initiate LD was positively skewed, suggesting that most respondents never (64.17%) or rarely had such an experience. Notably, item 5, concerning the frequency of successfully initiating LD, was endorsed only by those who have, at any time, attempted to initiate LD. Therefore, the analysis regarding this item was based on 67 respondents (36% of the sample). Distribution of the five frequency items of FILD are presented in **Figure 1**.

The notion that some individuals may have experienced lucidity during their childhood, but no longer in adulthood, which affected the design of the response scale, was supported; indeed, there were such respondents in each of the five frequency items (9.1, 13.4, 11.8, 11.8, and 6.4% of total responses, respectively, for the five items).

Items measuring LD intensity (items 6–10) were at nearly normal distribution. Most emotional valence variables (items 11–14) were nearly normally distributed, although the negative ending item (i.e., the item assessing the percentage of lucid dreams in which achieving lucidity led to a negative feeling) was somewhat positively skewed, suggesting that for most respondents, achieving lucidity led to a positive feeling, even though the lucid dream may have started out either positively or negatively. Notably, items 6–14 concerning LD intensity and emotional valence, were endorsed only by those who have, at any time, experienced some kind of LD. Therefore, the analysis was based on 142 respondents (76% of the sample).

The items measuring frequency of applying techniques for LD induction (e.g., keeping a dream diary, conducting reality checks, planning sleep time, thinking about LD before sleep) were mostly positively skewed, suggesting that most respondents who made at least one attempt to initiate LD did not frequently apply these LD induction techniques. Notably, Items 15–21 concerning the frequency of applying techniques for LD induction were endorsed only by those who have, at any time, attempted to initiate LD. Although 67 respondents answered in the affirmative to item 5 (deliberate LD initiation), only 64 completed the techniques section. Thus, analyses for this section were based on 64 respondents (34% of the sample).

Moving on to the follow-up daily dream diary phase, first of all we would like to mention that the 78 participants who chose to take part had very similar LD frequency data to the general sample. Specifically, 36% reported they had attempted to initiate LD in the past (compared to 35% in the general sample), 19% succeeded in doing so (compared to 20% in the general sample), 77% reported having LD in the past spontaneously (compared to 78% in the general sample), and 18% were non-lucid dreamers, of any kind (compared to 16% in the general sample). Thus, they seem to be a representative sub-sample of our total sample. Regarding LD frequency during the daily dream diary phase: 10 out of the 78 respondents (∼13%) reported the occurrence of LD during the 2-week period, and two of them, in fact, had two occurrences of LD (resulting in 12 lucid dreams overall in this study; notably, one participant reported that both of her lucid dreams were deliberately induced. The others reported that their lucidity was spontaneous).

#### Correlations Between the Trait FILD Items, and the Formation of Subscales

Correlations between FILD items spanning frequency, intensity and emotional valence are presented in **Table 1**, along with means and standard deviations. Additional items, which are less central, such as the frequency of applying techniques for LD induction, are detailed in the Supplementary Material, along with means and standard deviations (Supplementary Table S2). As can be seen in **Table 1**, all five items measuring LD frequencies of various types (items 1–5) were significantly correlated with one another (*r* = 0.24–0.79, *p* < 0.01). For example, spontaneous LD frequency was strongly correlated with deliberately induced LD, suggesting that those who tend to deliberately induce LD are the same individuals who tend to experience spontaneous LD. Given the medium to strong correlations between the five frequency items, we averaged them to create a composite lucidity frequency subscale. Cronbach’s alpha calculated for this subscale was an excellent 0.88. The scale was calculated based on all five frequency items. The 120 respondents for whom item 5 was not applicable (because they reported they never attempted to initiate LD on item 4), had N/A on that item, and their frequency scale score was calculated with the mean score of items 1–4. The score for the remaining 67 respondents was based on the mean of all 5 items, adding information about those respondent’s LD frequency (Ultimately, all 187 received a score on the frequency scale, based on the available data, and all analysis on the frequency scale in the study included the entire sample, although for some, it was calculated differently. Notably, using the mean score based only on items 1–4 for all 187 respondents was also examined; all results remained the same).

**Table 1:** Correlation coefficients, 95% Confidence Intervals for those coefficients, means, and standard deviations of FILD items.

Item	1	2	3	4	5	6	7	8	9	10	11	12	13	14
(1) Momentary	*–*													
(2) Prolonged	*0.60^∗∗∗^*	*–*												
	^[0.50,0.68]^													
(3) Spontaneous	*0.79^∗∗∗^*	*0.69^∗∗∗^*	*–*											
	^[0.73,0.84]^	^[0.61,0.76]^												
(4) Attempt	*0.27^∗∗∗^*	*0.32^∗∗∗^*	*0.24^∗∗^*	*–*										
	^[0.13,0.39]^	^[0.18,0.44]^	^[0.10,0.37]^											
(5) Success^a^	*0.54^∗∗∗^*	*0.74^∗∗∗^*	*0.49^∗∗∗^*	*0.68^∗∗∗^*	*–*									
	^[0.34,0.69]^	^[0.61,0.83]^	^[0.28,0.65]^	^[0.53,0.79]^										
(6) Confidence^b^	*0.20^∗^*	*0.25^∗∗^*	*0.28^∗∗^*	0.07	0.25	*–*								
	^[0.04,0.36]^	^[0.09,0.40]^	^[0.12,0.42]^	^[-0.10,0.23]^	^[-0.02,0.48]^									
(7) Activity^b^	0.03	*0.28^∗∗^*	0.09	*0.21^∗^*	0.08	*0.33^∗∗∗^*	*–*							
	^[-0.13,0.20]^	^[0.12,0.43]^	^[-0.08,0.25]^	^[0.04,-0.36]^	^[-0.19,0.34]^	^[0.17,0.47]^								
(8) Control^b^	0.10	*0.35^∗∗∗^*	0.13	*0.24^∗∗^*	*0.28^∗^*	*0.32^∗∗∗^*	*0.56^∗∗∗^*	*–*						
	^[-0.07,0.26]^	^[0.19,0.49]^	^[-0.04,0.30]^	^[0.07,-0.39]^	^[0.02,0.51]^	^[0.15,0.46]^	^[0.43,0.67]^							
(9) Length by scenes^b^	0.10	*0.38^∗∗∗^*	*0.25^∗∗^*	0.13	0.21	0.16	*0.28^∗∗^*	*0.48^∗∗∗^*	*–*					
	^[-0.06,0.27]^	^[0.30,0.57]^	^[0.05,0.37]^	^[0.04,-0.36]^	^[-0.02,0.48]^	^[-0.00,0.32]^	^[0.06,0.38]^	^[0.28,0.56]^						
(10) Length by seconds^b^	0.10	*0.45^∗∗∗^*	*0.21^∗^*	*0.20^∗^*	0.25	*0.19^∗^*	*0.22^∗∗^*	*0.43^∗∗∗^*	*0.54^∗∗∗^*	–				
	^[-0.07,0.26]^	^[0.23,0.52]^	^[0.08,0.40]^	^[-0.04,0.29]^	^[-0.06,0.45]^	^[0.00,0.32]^	^[0.12,0.43]^	^[0.34,0.60]^	^[0.40,0.64]^					
(11) Positive beginning^b^	-0.08	0.04	-0.07	0.04	0.03	0.02	0.15	0.16	0.05	0.02	*–*			
	^[-0.24,0.09]^	^[-0.13,0.21]^	^[-0.24,0.10]^	^[-0.13,0.21]^	^[-0.24,0.29]^	^[-0.15,0.19]^	^[-0.02,0.31]^	^[-0.01,0.32]^	^[-0.15,0.18]^	^[-0.12,0.22]^				
(12) Negative beginning^b^	0.12	-0.10	0.09	0.04	-0.02	0.04	-0.11	-0.11	-0.05	-0.02	-*0.70^∗∗∗^*	*–*		
	^[-0.05,0.28]^	^[-0.27,0.07]^	^[-0.09,0.25]^	^[-0.13,0.21]^	^[-0.29,0.26]^	^[-0.14,0.20]^	^[-0.27,0.06]^	^[-0.28,0.06]^	^[-0.19,0.16]^	^[-0.22,0.12]^	^[-0.78,-0.60]^			
(13) Positive ending^b^	0.13	*0.18^∗^*	*0.18^∗^*	0.07	0.09	*0.36^∗∗∗^*	0.15	*0.28^∗∗^*	*0.27^∗∗^*	*0.26^∗∗^*	*0.17^∗^*	-0.06	*–*	
	^[-0.04,0.29]^	^[0.01,0.34]^	^[0.01,0.33]^	^[-0.10,0.24]^	^[-0.18,0.35]^	^[0.21,0.50]^	^[-0.02,0.31]^	^[0.12,0.43]^	^[0.10,0.41]^	^[0.11,0.42]^	^[0.00,0.33]^	^[-0.23,0.11]^		
(14) Negative ending^b^	-0*.19^∗^*	-0.14	-*0.21^∗^*	0.06	0.01	-*0.28^∗∗^*	-0.01	-0.10	-*0.24^∗∗^*	-0.17	-0.08	*0.32^∗∗∗^*	-*0.60^∗∗∗^*	*–*
	^[-0.35,-0.02]^	^[-0.31,0.03]^	^[-0.37,-0.04]^	^[-0.12,0.23]^	^[-0.27,0.28]^	^[-0.43,-0.11]^	^[-0.18,0.16]^	^[-0.27,0.07]^	^[-0.33,0.00]^	^[-0.39,-0.07]^	^[-0.25,0.09]^	^[16,0.47]^	^[-0.70,-0.47]^	
*M*	2.45	1.71	2.38	0.90	1.61	1.70	1.51	1.01	1.19	1.48	49.80	46.79	63.68	28.77
*SD*	1.75	1.69	1.74	1.51	1.70	1.46	1.39	1.16	0.87	1.03	25.22	26.71	28.45	26.26

*Italics indicate statistically significant correlations. FILD = lucid dreaming questionnaire; momentary = frequency of momentary LD; prolonged = frequency of prolonged LD; Spontaneous = frequency of LD generated spontaneously; attempt = frequency of attempts to deliberately induce LD; success = frequency of successfully inducing LD; confidence = the extent to which the respondent feels confident that he or she is dreaming; active = the level of actively participating in dream events; control = the level of control over dream contents and events; length by scenes = evaluation of the duration of most LD by number of dream scenes; length by seconds = evaluation of the duration of most LD by seconds; positive beginning = the percentage of LD that starts as a positive dream (before lucidity onset); negative beginning = the percentage of LD that starts as a negative dream (before lucidity onset); positive ending = the percentage of LD in which achieving lucidity led to a positive feeling; negative ending = the percentage of LD in which achieving lucidity led to a negative feeling. ^∗^p < 0.05, ^∗∗^p < 0.01, ^∗∗∗^p < 0.001. ^*a*^Based only on N=67. ^*b*^Based only on N=142.*

The five items measuring the intensity of lucidity (items 6–10; endorsed by past or present lucid dreamers, 76% of the sample) were significantly correlated with one another (*r* = 0.19–0.56, *p* < 0.05), except for the correlation between confidence level and LD’s length by scenes, which was a non-significant statistical trend (*r* = 0.16, ns). Thus, we averaged them to create a composite lucidity intensity subscale. Cronbach’s alpha calculated for this subscale was a non-optimal 0.71.

As expected, the two items measuring emotional valence (positive and negative emotion) at the beginning of the lucid dream (item 11–12) were strongly inversely correlated (*r* = -0.70, *p* < 0.001), as were the two items measuring emotional valence after achieving lucidity (items 13–14) (*r* = -0.60, *p* < 0.001). Therefore, we reversed the negative items (items 12 and 14) and averaged the variables to create two subscales: emotional valence before, and emotional valence after, achieving lucidity. Cronbach’s alpha calculated for those subscales were 0.82 and 0.76, respectively. A high score in each of these scales indicates positive emotion.

The seven optional items measuring the frequency of applying techniques for LD induction (items 15–21; endorsed by LD initiators, 34% of the sample) also yielded significant correlations. Thus, the items were averaged to create a composite techniques subscale. Cronbach’s alpha calculated for this subscale was 0.72. A high score indicates frequent use of LD induction techniques.

#### Correlations Between FILD Subscales and Additional LD Measures

Inter-correlations between the subscales of the FILD are presented in **Table 2**. As can be seen in the table, higher intensity of LD is positively associated with higher frequency of LD as well as higher levels of positive emotion following lucidity onset. However, the magnitudes of these correlations are moderate. Notably, the two emotional valence subscales were only weakly correlated (*r* = 0.18, *p* < 0.05), suggesting that the emotional valence experienced prior to lucidity onset is not necessarily indicative of the emotional valence following lucidity onset (as mentioned above, our data suggest that emotional valence prior to gaining lucidity may be positive or negative, whereas emotional valence after gaining lucidity is usually positive). As may be expected, the techniques scale was correlated with the lucidity frequency scale.

**Table 2:** Correlations and 95% Confidence Intervals between subscales of the trait FILD, the binary daily LD variable, and the LD scale of the ISES.

Scale	1	2	3	4	5	6	7
(1) Lucidity frequency	*–*						
(2) Lucidity intensity^a^	*0.41^∗∗∗^*	*–*					
	*^[0.26,0.54]^*						
(3) Emotion after lucidity^a^	0.17	*0.34^∗∗∗^*	*–*				
	*^[0.00,0.32]^*	*^[0.18,0.48]^*					
(4) Emotion before lucidity^a^	-0.02	0.09	*0.18^∗^*	*–*			
	*^[^*^-^*^0.19,0.15]^*	*^[^*^-^*^0.08,0.25]^*	*^[0.01,0.34]^*				
(5) Techniques^b^	*0.38^∗∗^*	0.17	0.19	0.09	*–*		
	*^[0.14,0.57]^*	*^[^*^-^*^0.10,0.42]^*	*^[^*^-^*^0.08,0.44]^*	*^[^*^-^*^0.18,0.34]^*			
(6) Daily LD^c^	*0.38^∗∗∗^*	0.18	0.14	0.08	-0.06	–	
	*^[0.17,0.56]^*	*^[^*^-^*^0.08,0.41]^*	*^[^*^-^*^0.12,0.38]^*	*^[^*^-^*^0.18,0.33]^*	*^[^*^-^*^0.43,0.33]^*		
(7) ISES LD scale	*0.70^∗∗∗^*	*0.50^∗∗∗^*	*0.17^∗^*	-0.06	*0.45^∗∗∗^*	*0.37^∗∗^*	–
	*^[0.62,0.76]^*	*^[0.37,0.62]^*	*^[0.00,0.33]^*	*^[-0.22,0.11]^*	*^[0.22,0.62]^*	*^[0.07,0.48]^*	

*Italics indicate statistically significant correlations. FILD = lucid dreaming questionnaire; lucidity frequency = a scale of the five FILD frequency items (momentary, prolonged, spontaneous, attempt, and success); lucidity intensity = a scale of the five FILD intensity items (confidence, activity, control, length by seconds, and length by scenes); emotion before lucidity = a scale of the two FILD items measuring emotional valence before lucidity onset; emotion after lucidity = a scale of the two FILD items measuring emotional valence after lucidity onset; techniques = a scale of the seven FILD items measuring the frequency of applying techniques for LD induction; daily LD = a dichotomous variable assessing the occurrence or non-ocurrence of LD across the 14 days of the dream diary phase; ISES LD scale = the mean score of the three LD items of the Iowa sleep experiences survey. ^∗^p < 0.05, ^∗∗^p < 0.01, ^∗∗∗^p < 0.001. ^*a*^Based only on N = 142. ^*b*^Based only on N = 64. ^*c*^Based only on N = 78.*

**Table 2** also demonstrates preliminary support for the validity of the FILD, by presenting correlations of the subscales with additional indices representing LD frequency. First, the commonly used LD scale from the ISES was strongly correlated with the FILD lucidity frequency scale (*r* = 0.70, *p* < 0.001). The ISES LD scale was also correlated with the majority of the other FILD scales (*r* = 0.17–0.50, *p* < 0.05). Second, there was a significant biserial correlation between the lucidity frequency scale and the binary measure for LD occurrence (*r* = 0.38, *p* < 0.001), based on the daily diary.

#### Validation of FILD Using Receiver Operating Characteristic (ROC) Curves

We sought to determine a cutoff score for the FILD, for optimal sensitivity and specificity in identifying individuals who are likely to experience LD in a 2-week period, so that they could be easily screened in future studies. Thus, we used receiver operating characteristic (ROC) curves (Swets, 2014), to examine the extent to which the trait FILD frequency scale is able to differentiate the group of respondents who reported experiencing LD during the 2-week diary from those who did not.

The ROC analysis uses the association between sensitivity and specificity to derive an area under the curve (AUC), which indicates how well overall a continuous measure (the trait FILD frequency scale) may distinguish between case positive (LD, *n* = 10) and case negative (non-LD, *n* = 68) individuals (based on our binary daily diary LD frequency variable). A value of 0.50 of the AUC indicates chance level, and 1.0 indicates a perfect diagnostic tool. We determined a cutoff score by examining the coordinates of the ROC curve for the FILD frequency scale, in search of the value which would maximize both sensitivity (accurately identifying true positives) and specificity (accurately identifying true negatives).

**FIGURE 2:**
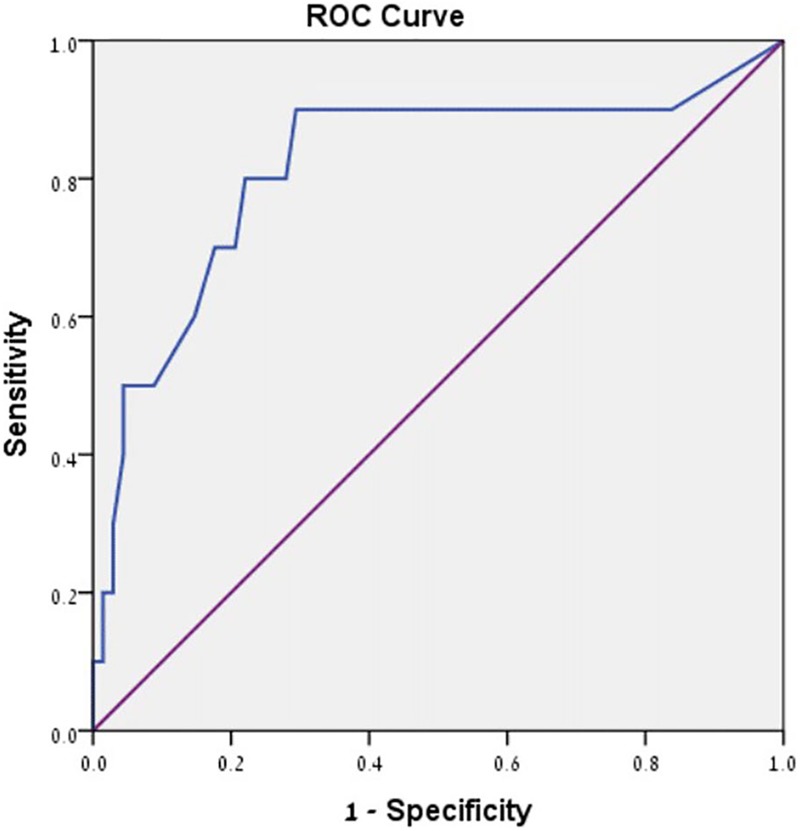
Receiver operating characteristic (ROC) curve for the lucidity frequency scale.

The FILD frequency scale had a good and statistically significant AUC value (*AUC* = 0.818, *SE* = 0.086, Asymptotic significance =< 0.01, *CI* = 0.649, 0.987), suggesting that this scale is capable of predicting who will experience LD within the 2-week period quite well. **Figure 2** presents the relationship between sensitivity and specificity, and **Table 3** indicates the sensitivity and specificity values for different possible values of the scale. As can be seen in the table, the optimal cutoff value for the lucidity frequency scale seems to be a score of 2.23. When using the criterion that only those scoring 2.23 or above will be classified as positive for LD, sensitivity is 90% (meaning that 9 out of 10 frequent lucid dreamers in this study would have been classified as such) and specificity is 71% (meaning that 48 of the 68 non-lucid dreamers in this study would have been correctly classified as such). An alternative cutoff value, for increased specificity over sensitivity, is 2.45; when using that criterion, sensitivity is 80% (meaning that 8 out of 10 frequent lucid dreamers in this study would have been classified as such) and specificity is 78% (meaning that 53 of the 68 non-lucid dreamers in this study would have been correctly classified as such).

**Table 3:** Coordinates of the ROC curve for the lucidity frequency scale.

Lucidity frequency cutoff score	Sensitivity	Specificity
-1.0000	100.0%	0.0%
0.1000	90.0%	16.2%
0.2250	90.0%	19.1%
0.3750	90.0%	20.6%
0.6250	90.0%	26.5%
0.7750	90.0%	29.4%
0.9000	90.0%	32.4%
1.1250	90.0%	36.8%
1.3250	90.0%	44.1%
1.4500	90.0%	45.6%
1.5500	90.0%	51.5%
1.6750	90.0%	52.9%
1.7750	90.0%	55.9%
1.9000	90.0%	57.4%
2.1000	90.0%	67.6%
2.2250	90.0%	70.6%
2.3250	80.0%	72.1%
2.4500	80.0%	77.9%
2.6250	70.0%	79.4%
2.7750	70.0%	82.4%
2.9000	60.0%	85.3%
3.1000	50.0%	91.2%
3.2250	50.0%	95.6%
3.3250	40.0%	95.6%
3.5000	30.0%	97.1%
3.6750	20.0%	97.1%
3.7750	20.0%	98.5%
3.9000	10.0%	98.5%
4.4000	10.0%	100.0%
5.8000	0.0%	100.0%

### Part II: Relationships With Psychopathology and Sleep (*H2, H3*)

#### Correlations of FILD Scales With Distress and Sleep Quality Measures (*H2*)

Correlations between the FILD scales and stress, psychopathology and sleep quality measures are presented in **Table 4** (means and standard deviations of psychopathology measures are also included in the table). For frequency and intensity scales of LD, correlations with individual items are also included, as these scales encompass several domains and thus are also a focus of interest. Notably, we considered presenting partial correlations, controlling for age and gender, but the correlations between the demographic variables and the rest of the study variables were mostly non-significant, with only a few exceptions. Specifically, out of all the FILD items as well as sleep and distress measures at Times 1 and 2, gender was correlated only with positive and negative emotion before lucidity onset (*r* = 0.24 and -0.25, respectively), indicating that women tended to have less positive emotion and more negative emotion, compared to men, prior to lucidity onset. In addition, age correlated solely with Time 1 dissociation (*r* = -0.21). Nevertheless, controlling for age and gender was examined, and results remained the same.

**Table 4:** Correlation coefficients and 95% Confidence Intervals for those coefficients, representing the relationships between FILD items with stress, psychopathology, and sleep problems.

Measure	PSS T1	BDI T1	BAI T1	MOCI T1	DES T1	MIS T1	GSAQ T1
Item 1 – Momentary frequency	0.01	-0.08	-0.01	-0.01	0.01	0.09	0.08
	*^[^*^-^*^0.14,0.15]^*	*^[^*^-^*^0.22,0.07]^*	*^[^*^-^*^0.15,0.13]^*	*^[^*^-^*^0.15,0.14]^*	*^[^*^-^*^0.13,0.16]^*	*^[^*^-^*^0.05,0.23]^*	*^[^*^-^*^0.06,0.22]^*
Item 2 – Prolonged frequency	-0.03	-0.06	-0.05	-*0.14^∗^*	-0.04	0.06	0.12
	*^[^*^-^*^0.17,0.12]^*	*^[^*^-^*^0.21,0.08]^*	*^[^*^-^*^0.20,0.09]^*	*^[^*^-^*^0.28,0.00]^*	*^[^*^-^*^0.18,0.11]^*	*^[^*^-^*^0.08,0.20]^*	*^[^*^-^*^0.02,0.26]^*
Item 3 – Spontaneous frequency	-0.07	-0.12	-0.04	-0.08	-0.04	0.02	0.06
	*^[^*^-^*^0.21,0.08]^*	*^[^*^-^*^0.26,0.03]^*	*^[^*^-^*^0.19,0.10]^*	*^[^*^-^*^0.22,0.07]^*	*^[^*^-^*^0.19,0.10]^*	*^[^*^-^*^0.12,0.16]^*	*^[^*^-^*^0.08,0.21]^*
Item 4 – Attempt frequency	*0.21^∗∗^*	0.14	0.10	*0.15^∗^*	*0.16^∗^*	0.14	*0.23^∗∗^*
	*^[0.06,0.34]^*	*^[^*^-^*^0.01,0.28]^*	*^[^*^-^*^0.05,0.24]^*	*^[0.01,0.29]^*	*^[0.01,0.30]^*	*^[^*^-^*^0.01,0.28]^*	*^[0.08,0.36]^*
Item 5 – Success frequency^a^	0.00	-0.04	0.02	-0.04	0.12	0.14	0.20
	*^[^*^-^*^0.24,0.24]^*	*^[^*^-^*^0.27,0.21]^*	*^[^*^-^*^0.22,0.26]^*	*^[^*^-^*^0.28,0.20]^*	*^[^*^-^*^0.12,0.35]^*	*^[^*^-^*^0.10,0.37]^*	*^[^*^-^*^0.04,0.42]^*
**Lucidity frequency**	**0.01**	-**0.06**	-**0.01**	-**0.04**	**0.03**	**0.10**	***0.15^∗^***
	***^[^***^-^***^0.13,0.16]^***	***^[^***^-^***^0.20,0.09]^***	***^[^***^-^***^0.15,0.14]^***	***^[^***^-^***^0.18,0.10]^***	***^[^***^-^***^0.12,0.17]^***	***^[^***^-^***^0.04,0.24]^***	***^[0.00,0.28]^***

Item 6 – Confidence^b^	-*0.25^∗∗^*	-*0.19^∗^*	-*0.25^∗∗^*	-0.16	-0.08	-0.12	-0.10
	*^[^*^-^*^0.40,^*^-^*^0.09]^*	*^[^*^-^*^0.35,^*^-^*^0.03]^*	*^[^*^-^*^0.40,^*^-^*^0.09]^*	*^[^*^-^*^0.32,0.00]^*	*^[^*^-^*^0.24,0.09]^*	*^[^*^-^*^0.28,0.05]^*	*^[^*^-^*^0.26,0.07]^*
Item 7 – Activity^b^	-0.10	-0.04	-0.16	0.07	0.02	-0.03	-0.06
	*^[^*^-^*^0.26,0.07]^*	*^[^*^-^*^0.21,0.13]^*	*^[^*^-^*^0.31,0.01]^*	*^[^*^-^*^0.10,0.23]^*	*^[^*^-^*^0.15,0.18]^*	*^[^*^-^*^0.19,0.14]^*	*^[^*^-^*^0.23,0.11]^*
Item 8 – Control^b^	-*0.19^∗^*	-*0.20^∗^*	-*0.24^∗∗^*	-*0.21^∗^*	-0.03	-0.09	-0.06
	*^[^*^-^*^0.35,^*^-^*^0.02]^*	*^[^*^-^*^0.36,^*^-^*^0.04]^*	*^[^*^-^*^0.39,^*^-^*^0.07]^*	*^[^*^-^*^0.36,^*^-^*^0.04]^*	*^[^*^-^*^0.20,0.14]^*	*^[^*^-^*^0.25,0.08]^*	*^[^*^-^*^0.22,0.11]^*
Item 9 – Length by seconds^b^	-*0.17^∗^*	-0.11	-0.10	-*0.17^∗^*	-0.08	-0.09	0.01
	*^[^*^-^*^0.33,^*^-^*^0.01]^*	*^[^*^-^*^0.27,0.06]^*	*^[^*^-^*^0.26,0.07]^*	*^[^*^-^*^0.33,0.00]^*	*^[^*^-^*^0.24,0.09]^*	*^[^*^-^*^0.25,0.08]^*	*^[^*^-^*^0.16,0.17]^*
Item 10 – Length by scenes^b^	-*0.22^∗∗^*	-0.16	-0.09	-*0.24^∗∗^*	-0.07	-0.06	-0.03
	*^[^*^-^*^0.37,^*^-^*^0.05]^*	*^[^*^-^*^0.32,0.01]^*	*^[^*^-^*^0.25,0.08]^*	*^[^*^-^*^0.39,^*^-^*^0.08]^*	*^[^*^-^*^0.23,0.10]^*	*^[^*^-^*^0.22,0.11]^*	*^[^*^-^*^0.19,0.14]^*
**Lucidity intensity^b^**	-***0.22^∗∗^***	-***0.18^∗^***	-***0.24^∗∗^***	-**0.16**	-**0.06**	-**0.13**	-**0.04**
	***^[^***^-^***^0.37,^***^-^***^0.06]^***	***^[^***^-^***^0.33,^***^-^***^0.01]^***	***^[^***^-^***^0.39,^***^-^***^0.08]^***	***^[^***^-^***^0.32,0.00]^***	***^[^***^-^***^0.22,0.11]^***	***^[^***^-^***^0.28,0.04]^***	***^[^***^-^***^0.21,0.13]^***

**Emotion before lucidity^b^**	-***0.26^∗∗^***	-***0.22^∗^***	-***0.30^∗∗∗^***	^-^**0.15**	-**0.06**	-**0.02**	-***0.23^∗∗^***
	***^[^***^-^***^0.41,^***^-^***^0.10]^***	***^[^***^-^***^0.37,^***^-^***^0.05]^***	***^[^***^-^***^0.44,^***^-^***^0.14]^***	***^[^***^-^***^0.31,0.02]^***	***^[^***^-^***^0.23,0.11]^***	***^[^***^-^***^0.18,0.15]^***	***^[^***^-^***^0.38,^***^-^***^0.06]^***
**Emotion after lucidity^b^**	-***0.38^∗∗∗^***	-***0.21^∗^***	-***0.18^∗^***	-**0.16**	-**0.11**	-**0.06**	-**0.12**
	***^[^***^-^***^0.51,^***^-^***^0.23]^***	***^[^***^-^***^0.37,^***^-^***^0.05]^***	***^[^***^-^***^0.33,^***^-^***^0.01]^***	***^[^***^-^***^0.32,0.01]^***	***^[^***^-^***^0.28,0.06]^***	***^[^***^-^***^0.22,0.11]^***	***^[^***^-^***^0.28,0.05]^***

**Techniques^c^**	**0.21**	***0.35^∗∗^***	**0.15**	***0.26^∗^***	***0.32^∗^***	***0.34^∗∗^***	***0.37^∗∗^***
	***^[^***^-^***^0.03,0.44]^***	***^[0.11,0.55]^***	***^[^***^-^***^0.10,0.38]^***	***^[0.02,0.48]^***	***^[0.07,0.52]^***	***^[0.10,0.54]^***	***^[0.14,0.57]^***

**ISES LD scale**	**0.07**	**0.03**	**0.04**	-**0.02**	**0.12**	***0.17^∗^***	***0.26^∗∗∗^***
	***^[^***^-^***^0.07,0.21]^***	***^[^***^-^***^0.12,0.17]^***	***^[^***^-^***^0.10,0.18]^***	***^[^***^-^***^0.16,0.13]^***	***^[^***^-^***^0.03,0.26]^***	***^[0.02,0.30]^***	***^[0.12,0.39]^***

*M*	14.68	10.00	11.46	9.19	12.87	5.99	1.55
*SD*	6.60	8.05	9.58	5.04	11.62	5.56	0.26

*Italics indicate statistically significant correlations. Bold indicates scales. FILD = lucid dreaming questionnaire; momentary = frequency of momentary LD; prolonged = frequency of prolonged LD; spontaneous = frequency of LD generated spontaneously; attempt = frequency of attempts to deliberately induce LD; success = frequency of successfully inducing LD; lucidity frequency = a scale of the five frequency items (momentary, prolonged, spontaneous, attempt, and success); confidence = the extent to which the respondent feels confident that he or she is dreaming; activity = the level of actively participating in dream events; control = the level of control over dream contents and events; length by scenes = evaluation of the duration of most lucid dreams by number of dream scenes; length by seconds = evaluation of the duration of most lucid dreams by seconds; lucidity intensity = a scale of the five intensity items (confidence, activity, control, length by seconds, and length by scenes); emotion before lucidity = a scale of the FILD, two items measuring emotional valence before lucidity onset; emotion after lucidity = a scale of the FILD, two items measuring emotional valence after lucidity onset; techniques = a scale of the FILD, seven items measuring the frequency of applying techniques for LD induction; ISES LD scale = the three LD items of the Iowa sleep experiences survey; PSS = Perceived Stress Scale; BDI = Beck Depression Inventory; BAI = Beck Anxiety Inventory; MOCI = Maudsley Obsessive Compulsive Inventory; DES = revised Dissociative Experiences Scale; MIS = Magical Ideation Scale; GSAQ = Global Sleep Assessment Questionnaire. ^∗^p < 0.05, ^∗∗^p < 0.01, ^∗∗∗^p < 0.001. ^*a*^Based only on N = 67. ^*b*^Based only on N = 142. ^*c*^Based only on N = 64. Below are means and standard deviations for stress, psychopathology, and sleep problems.*

No significant correlations were found between the lucidity frequency scale and the psychopathology and stress measures. However, frequent LD was weakly associated with sleep problems (notably, we explored the possibility that the lack of relationships with psychopathology stem from our inclusion of an “only at childhood” response in the frequency scale. However, removing this response, along with the respondents who endorsed it, did not alter the results). Despite the general lack of associations with psychopathology for the total scale, inspection of individual items from this scale shows that deliberate LD induction was weakly positively associated with sleep problems, OC symptoms, dissociation, and stress.

The lucidity intensity scale was significantly inversely correlated with depression, anxiety and stress. When inspecting individual item correlations, it seems that control and confidence in lucidity are central items in determining these relations, whereas activity is not. In addition, the longer lucid dreams the individual reports, the less likely that individual is to report stress and OC symptoms (the negative relation with OC is also evident through item 2 of the frequency scale, assessing prolonged frequency). No significant correlation was found between lucidity intensity and sleep quality. Notably, the ISES LD scale (Watson, 2001) was correlated solely with schizotypy and sleep problems.

It is important to consider that the correlations between the FILD intensity scale and psychopathology are limited to those who have had at least one lucid dream, and thus responded to the intensity items (76% of the sample). However, we wished to also compare psychopathology levels of the non-lucid dreamers (14% of the sample; the remaining 10% of the sample reported experiencing some kind of LD in the frequency items of the FILD, but did not complete the intensity items, and thus were not included in this analysis), to psychopathology levels of high-intensity lucid dreamers (37.5% of the sample, those scoring over the median of the intensity score, which was 1.20). Thus, we performed ANOVA models with the two groups as the independent variable, and each psychopathology variable as the dependent variable; these models did not reach statistical significance, suggesting that high-intensity lucid dreamers were not more resilient (or less distressed) than non-lucid dreamers.

Positive emotional valence in LD, both before and after lucidity onset, was associated with lower levels of psychopathological symptoms (depression, anxiety, and stress), and positive valence before lucidity onset was also associated with less sleep problems.

Lastly, the frequency of applying techniques for deliberate LD induction was positively correlated with depression, OC symptoms, dissociation, schizotypy, and sleep problems.

#### A Prospective-Longitudinal Model for LD and Psychopathology (H3)

Multiple linear regression analysis with a prospective-longitudinal design was utilized to explore the possible longitudinal effect of LD frequency on psychopathology symptoms. Specifically, predicting Time 2 psychopathology while controlling for Time 1 psychopathology is tantamount to predicting change. In each regression model, change over time in psychopathological symptoms was predicted by either spontaneous or deliberately induced LD frequency. Induced LD frequency positively predicted a longitudinal increase in dissociation symptoms, when using the general dissociation score (*b* = 1.47, *SE* = 0.44, β = 0.26, *t* = 3.31, *p* < 0.01, *CI* = 0.58,2.35) as well as with using each of the measure’s three subscales – depersonalization (*b* = 1.09, *SE* = 0.46, β = 0.25, *t* = 2.38, *p* < 0.05, *CI* = 0.17,2.0), amnesia (*b* = 1.03, *SE* = 0.51, β = 0.22, *t* = 2.01, *p* < 0.05, *CI* = 0.01,2.05) and absorption (*b* = 1.37, *SE* = 0.63, β = 0.17, *t* = 2.19, *p* < 0.05, *CI* = 0.12,2.62). Induced LD frequency also predicted a longitudinal increase in schizotypy (*b* = 0.47, *SE* = 0.23, β = 0.14, *t* = 2.06, *p* < 0.05, *CI* = 0.02,0.92) in the subsequent 2-month period. Notably, such effects did not emerge with the spontaneous LD frequency item or with the ISES LD scale.

## Discussion

The current study explored three main hypotheses, pertaining to the development of the FILD, the cross-sectional relationships of LD with psychopathology, and the possible longitudinal effect of LD on psychopathology. We will discuss each of these in turn.

Our first aim in this study was to develop and validate an expansive LD questionnaire, assessing several characteristics of LD and presenting an alternative to the relative simple measurements used to assess LD in most previous studies. Accordingly, our first hypothesis (*H1*) was that the FILD will demonstrate adequate validity and reliability. The study offers preliminary evidence suggesting that the FILD is indeed a valid and reliable measure for assessing LD. Correlations between items seemed to support our differentiation between *frequency* and *intensity*. Spontaneous and deliberately induced LD frequencies were highly associated, as were momentary and prolonged LD frequencies. They may be causally related; for example, attempting to deliberately induce LD may evoke spontaneous LD frequency. The levels of control, confidence, activity, and length of LD, were mostly associated with each other, together forming a novel scale of the intensity of LD. This is consistent with a recent finding showing that dream insight (a measure which is somewhat parallel to the level of confidence in lucidity) and dream control are highly correlated (Voss et al., 2013). Although the frequency scale was positively associated with the intensity scale, and the latter was associated with the positive affect following lucidity scale, the magnitudes of these correlations were moderate, supporting the notion that they are not identical constructs.

In terms of reliability, the FILD’s frequency subscale had a very good Cronbach’s alpha value, whereas the other subscales had alphas ranging from acceptable, yet somewhat non-optimal (intensity, emotional valence following lucidity onset, and induction techniques use), to good (emotional valence before lucidity onset). Future studies should examine reliabilities within larger and more heterogeneous samples, as well as examine temporal stability of these scales. In terms of validity, the FILD was strongly associated with a common LD frequency measure, namely, the LD subscale of the ISES (Watson, 2001). Validity was additionally strengthened by the relation between the FILD frequency subscale and the daily diary occurrence of LD, a near-experience measure which is probably less affected by memory distortion and less biased than retrospective reports. Moreover, we have specified optimal cutoff values for the FILD frequency scale, which will help future LD research screen and identify frequent lucid dreamers for their longitudinal studies. Researchers are advised to use a cutoff of 2.23 for an inclusive, optimal-sensitivity criterion, or a cutoff of 2.45 for a balanced sensitivity-specificity criterion.

Our second aim in this study was to explore the associations of the various LD characteristics with psychopathological symptoms. Our second hypothesis (*H2*), suggested that lucidity characterized by high intensity and positive affect will relate to fewer psychopathological tendencies. This hypothesis was supported, as intensity and emotional valence were inversely related to distress variables. Notably, high-intensity lucid dreamers were not more resilient (i.e., less distressed) as compared with non-lucid dreamers; however, they were more resilient compared with low-intensity lucid dreamers. Taken together with the finding that the frequency scale was unrelated to psychopathology variables, this suggests that merely having awareness that one is dreaming does not guarantee enhanced resilience or well-being, nor does it indicate psychopathological distress. However, to the extent that one is aware of dreaming, the characteristics of this awareness do carry significance as to the individual’s tendencies for psychopathological symptoms. Specifically, LD which are accompanied by a sense of control and confidence in lucidity, and which evoke positive affect are related to lesser distress than LD in which the dreamer does not control dream events, is uncertain of the state of dreaming, and experiences negative affect. This is compatible with the idea that arousal within the sleeping consciousness is related to distress when it is experienced as intrusive, but not when it is experienced as controlled (Soffer-Dudek, 2017a).

The finding that intensity, but not frequency, is inversely related to psychopathology underscores the importance of assessing LD as a complex phenomenon, rather than relying on single LD scores, as was done in most previous studies. Results of the present study may serve as the explanation for previous conflicting findings which were reviewed in the introduction section, according to which, LD was positively related to psychopathology in some studies (e.g., Watson, 2001; Taitz, 2011) but not in others (e.g., Knox and Lynn, 2014; van Heugten–van der Kloet et al., 2014). These previous conflicting findings may stem from the lack of distinction between LD frequency and intensity. Our findings support the idea that some individuals experience awareness in dreams but with little sense of control and with negative affect. Indeed, in one study, positive emotion differed between lucid and non-lucid dreams, whereas negative emotion did not (Voss et al., 2013). The present study provides initial evidence against the prevailing opinion that LD is necessarily positive and emotionally beneficial (Tholey, 1988; LaBerge, 2014). Indeed, the associations found between LD and well-being (Snyder and Gackenbach, 1988; Gruber et al., 1995) and between LD and psychological resilience (Soffer-Dudek et al., 2011b), may have been rooted in reports of intense and positively toned LD experiences.

Finally, our third hypothesis (*H3*), suggested that deliberate LD induction may result in a longitudinal increase in dissociative symptoms. This hypothesis was supported; deliberately induced LD frequency (but not spontaneous LD frequency) significantly predicted a future increase in both dissociation and schizotypy symptoms, over a 2-month period. This supports the idea that popular LD induction techniques (e.g., reality testing, the reflection technique) may impair sleep-wake boundaries and thus induce symptoms characteristic of psychopathologies in which differentiation between reality and fantasy is impaired, such as derealization. To the best of our knowledge, this is the first study to explore, and demonstrate, a potential long-term risk following the use of LD induction techniques. This longitudinal finding is strengthened by the cross-sectional findings, according to which, higher use of induction techniques was associated with various psychopathological symptoms (dissociation, schizotypy, depression, and OC symptoms), as well as with increased sleep problems. Although causal conclusions should be considered with caution regarding the cross-sectional findings, it is possible that LD induction causes disturbed sleep, as some of the common initiation techniques actually require scheduled awakenings in the middle of the night, in turn possibly causing affective symptoms. Alternatively, OC or depressed individuals may be more prone to attempt to initiate LD, as their sleep is already disturbed (e.g., Johnson et al., 2006). The present findings may shed light on the theoretical stance that LD are a part of an “unusual cognitions” continuum (Watson, 2001); specifically, it is possible that higher associations of LD with dissociation and schizotypy, compared with general psychological distress, may be rooted not in LD *per se*, but in the use of LD induction techniques.

These findings are especially important in light of the growing interest in LD induction. In our sample, over a third of respondents reported that they had tried to induce LD at least once. Future research on LD in young adults or student populations should take into account that LD has become a popular and well-known phenomenon; previous LD experience should be assessed and controlled for in such studies.

Several limitations of the current study should be noted: (1) the data in this study relied exclusively on self-report measures, and thus shared method variance may have inflated the associations between the study constructs. For example, people who tend to report that they experience highly negative emotion regarding their waking life may tend to also report highly negative emotion, or decreased controllability, in their dreams, due to a reporting bias rather than a true relation. Validity of psychopathology measures would have been strengthened by assessment conducted by a mental health professional. In addition, it is yet unclear whether people may validly report subtleties of dream experiences, such as length of LD; although, it seems that time perception in LD is similar to that of waking (LaBerge, 2000). Still, future studies should utilize sleep laboratory objective assessments and reports proximal to the LD in order to fully validate the FILD and its relation with psychopathology. However, a notable strength of this study is the reliance on daily diaries in addition to retrospective questionnaires, which measure LD in proximity to the experience and are thus less biased. (2) The reliance on a non-clinical, mostly female, sample of university students restricts the ability to generalize these results to other populations and specifically to clinical-level psychopathology. However, it is important to note that the definition of the sample as non-clinical does not mean that none of the participants had clinical-range symptoms and disorders. In fact, because psychopathology levels found among college students are similar to the prevalence in same-aged non-students (Hunt and Eisenberg, 2010), this sample probably represents various levels of mental health, and results may be generalized to same-aged community samples. This is supported by inspecting means of psychopathology measures presented in **Table 4**, which were similar to norms for community populations (for example, the BDI in this sample had an average of 10, similar to a figure of 10.6 reported for a community sample spanning 7,500 Dutch respondents; Roelofs et al., 2013, and higher than a figure of 6.25 reported for an Australian community sample; Crawford et al., 2011. Similarly, our mean of 11.46 for the BAI was similar or higher than norms reported for community samples in the literature, such as 6.16; Crawford et al., 2011, 6.6; Gillis et al., 1995; and 11.54; Osman et al., 1993). Importantly, socioeconomic level was not measured, and it also may have had a psychopathological impact on the participants in this study that was not taken into account.^6^ (3) Several of the effects and correlations were quite small in magnitude, and also, some of them relied only on part of the participants (e.g., correlations with induction techniques), resulting in a smaller sample. Thus, they should be considered with caution and replicated in future studies. Notably, however, several of the findings in this study replicated across different psychopathology measures, or replicated from a cross-sectional design to a longitudinal one; thus, the findings do not seem to be random. (4) Many of the findings in the current study relied on cross-sectional analyses, precluding conclusions regarding causality. However, we also showed directionality by demonstrating an increase in symptoms 2 months after reports of high LD frequency.

Nevertheless, the study’s limitations should be weighed in the context of its strengths and contributions. The fine resolution in which the different LD components were examined using the FILD enabled a better understanding of the aspects of LD that may be positive and beneficial to one’s mental health, versus those which may be detrimental. This is an important contribution to the literature, which should invariably make the distinction between spontaneous and deliberately induced LD, and between frequency and intensity of LD. The study suggests a solution for the current inconsistencies in LD literature, in which relations with psychopathology are unclear; it seems that LD intensity and emotional valence, rather than mere occurrence, are more relevant to the exploration of relations with psychopathology. This is also the first study to report a possible negative long-term effect of LD induction. Our longitudinal findings, which pertain to change within individuals, cannot be alternatively explained by individual differences between respondents (e.g., those who choose to initiate LD are more distressed individuals). This is an important contribution of the present investigation, suggesting that the application of reality monitoring techniques in order to achieve LD might be detrimental to one’s normal, healthy ability to differentiate reality from fantasy and waking from dreaming. Clinicians and researchers using LDT should take this into account and examine whether the possible benefits outweigh the risks, such as possible interferences to sleep, mood, reality monitoring and perception, and to a disruption or discontinuity in the normal integration of consciousness. In addition, when using LDT, researchers should mind and attempt to increase intensity and positive emotional valence of LD, and not only its frequency. It has been previously suggested that the mere feeling of mastery and the idea of being able to control the nightmare that the participants gained while undergoing LDT was beneficial in nightmare reduction, even if they failed to become lucid due to a relatively brief intervention (Gavie and Revonsuo, 2010). A future emphasis on the aspect of control in LDT along with other positive aspects identified in the current study, and careful consideration of the negative aspects, may inform LDT procedures as well as clarify the mechanism of change for this intervention.

## Ethics Statement

All subjects gave written informed consent in accordance with the Declaration of Helsinki. The protocol was approved by the Human Subjects Research Committee of Ben-Gurion University.

## Author Contributions

LA and NS-D were responsible for the conception, literature review, data collection, data analysis, and writing of this manuscript. This research is part of LA’s graduate studies thesis, conducted under the supervision of NS-D.

## Conflict of Interest Statement

The authors declare that the research was conducted in the absence of any commercial or financial relationships that could be construed as a potential conflict of interest.

## Footnotes

1 Notably, in this study, dream awareness and an observer stance were encouraged as part of nightmare rescripting, but LD was not straight forwardly discussed as part of the treatment.

2 Notably, this study was planned in parallel to the publication of the LuCiD questionnaire by Voss et al. (2013), thus we were unaware of that questionnaire at the time and developed a measure of our own.

3 This ratio is representative of the male–female ratio in the Psychology Department.

4 It is justifiable to use ordinal-level response-scales with parametric statistics when there are enough categories (e.g., Tabachnick and Fidell, 2007; Norman, 2010; Rhemtulla et al., 2012).

5 We also assessed daily psychopathology and sleep quality, but we do not use those data because of the sparsity of daily LD experiences (i.e., exploring within-subjects associations between LD and psychopathology would have required relying on a small sample, questioning generalizability).

6 A reviewer also suggested that since the sample consists mainly of women, female sex hormones may have influenced the results (e.g., the relation between sleep quality and psychopathology); unfortunately, we did not assess and control for menstrual/hormonal cycle.

## Abbreviations

FILD: Frequency and Intensity Lucid Dreaming questionnaire
LD: lucid dreaming
LDT: lucid dreaming treatment
OC: obsessive-compulsive
