# Supplementary material for: Lucid Dreaming: Intensity, But Not Frequency, Is Inversely Related to Psychopathology
Source: Front Psychol. 2018 Mar 22;9:384. doi: 10.3389/fpsyg.2018.00384 (PMC5875414; doi:10.3389/fpsyg.2018.00384)
Supplement: Supplementary file 1 [file Table_1.docx]

Supplementary Material

Lucid Dreaming:

How frequency and intensity are related to psychopathology

Liat Aviram^1^, Nirit Soffer-Dudek^1*^

^1^The Consciousness and Psychopathology laboratory, Ben-Gurion University of the Negev, Department of Psychology, Beer-Sheva, Israel.

*** Correspondence:**Dr. Nirit Soffer-Dudek

[soffern@bgu.ac.il](mailto:soffern@bgu.ac.il)

**Section S1**

**Frequency and Intensity Lucid Dreaming Questionnaire (FILD)**

Please read the following description carefully:

*In a lucid dream, the dreamer is aware of the fact that he or she is dreaming, within the dream state. That awareness can last for a brief moment (for example, the realization that one is dreaming followed by an immediate awakening) or for a longer period of time (i.e., prolonged awareness within the dream). In some cases, after achieving lucidity, the dreamer may also control the content of the dream and voluntarily alter the dream events. Notably, some people experience lucid dreams spontaneously (i.e., lucidity occurs without deliberately attempting to achieve it), while others experience initiated lucidity (i.e., lucidity occurs following the deliberate implementation of lucidity induction techniques).*

The following questions refer to the extent to which you may have experienced the phenomenon of lucid dreaming.

**Section A**

In the following questions, please select the answer most accurate according to your own experiences:

1. Try to estimate the frequency with which you experience momentary lucidity in a dream, following by an immediate awakening.

- I have never experienced momentary lucidity within a dream.
- I have experienced it during childhood but not in adulthood.
- I experience it less than once a year.
- I experience it once or twice a year.
- I experience it 3-11 times a year.
- I experience it once or twice a month.
- I experience it 3-4 times a month.
- I experience it twice a week or more.

1. Try to estimate the frequency with which you experience prolonged lucidity in a dream.

- I have never experienced prolonged lucidity within a dream.
- I have experienced it during childhood but not in adulthood.
- I experience it less than once a year
- I experience it once or twice a year.
- I experience it 3-11 times a year.
- I experience it once or twice a month.
- I experience it 3-4 times a month.
- I experience it twice a week or more.

1. Try to estimate the frequency with which you experience lucidity (whether momentary or prolonged) in a dream spontaneously (i.e., without attempting to deliberately achieve lucidity).

- I have never experienced spontaneous lucidity within a dream.
- I have experienced it during childhood but not in adulthood.
- I experience it less than once a year
- I experience it once or twice a year.
- I experience it 3-11 times a year.
- I experience it once or twice a month.
- I experience it 3-4 times a month.
- I experience it twice a week or more.

1. Try to estimate the frequency with which you attempt to initiate lucidity in a dream deliberately. Note that this question refers to the frequency of attempts, and not to the frequency of attempts that were successful.

- I have never attempted to initiate lucidity in a dream deliberately.
- I have attempted it during childhood but not in adulthood.
- I attempt it less than once a year
- I attempt it once or twice a year.
- I attempt it 3-11 times a year.
- I attempt it once or twice a month.
- I attempt it 3-4 times a month.
- I attempt it twice a week or more.

1. Try to estimate the frequency with which you successfully achieve lucidity in a dream due to a deliberate attempt. ^^[[1]](#footnote-1)^^

- I have never successfully achieved lucidity in a dream due to a deliberate attempt.
- I have successfully achieved lucidity during childhood but not in adulthood.
- I successfully achieve lucidity less than once a year
- I successfully achieve lucidity once or twice a year.
- I successfully achieve lucidity 3-11 times a year.
- I successfully achieve lucidity once or twice a month.
- I successfully achieve lucidity 3-4 times a month.
- I successfully achieve lucidity twice a week or more.

**Section B**

This section of the questionnaire is designated only for those who experienced some kind of lucid dreaming, whether spontaneous or initiated, momentary or prolonged, at least once. If you answered “never” in items 1 and 2, you may skip the current section.

In the following questions, please select the answer most accurate according to your experiences:

1. Try to estimate the percentage of lucid dreams in which you experienced **confidence** in your lucidity (a state in which you have a clear understanding that you are dreaming), versus **uncertainty** in your lucidity (a state in which you ask yourself whether you are dreaming or not).

- I’m uncertain in my lucidity in the vast majority of my lucid dreams (roughly 80%-100%).
- I’m uncertain in my lucidity in most of my lucid dreams (roughly 60%-70%).
- I’m uncertain in my lucidity in roughly 50% of my lucid dreams and confident in my lucidity in roughly 50% of my lucid dreams.
- I’m confident in my lucidity in most of my lucid dreams (roughly 60%-70%).
- I’m confident in my lucidity in the vast majority of my lucid dreams (roughly 80%-100%).

1. Try to estimate the percentage of lucid dreams in which you take an **active stance** (set goals and actions and attempt to execute them) versus a **passive stance** (continue observing the dream as in a regular dream) after achieving lucidity.

- I choose a passive stance in the vast majority of my lucid dreams (roughly 80%-100%).
- I choose a passive stance in most of my lucid dreams (roughly 60%-70%).
- I choose a passive stance in roughly 50% of my lucid dreams and an active stance in roughly 50% of my lucid dreams.
- I choose an active stance in most of my lucid dreams (roughly 60%-70%).
- I choose an active stance in the vast majority of my lucid dreams (roughly 80%-100%).

1. Try to estimate the percentage of lucid dreams in which you are able to **control** and manipulate the dream content and events volitionally, versus a sense that events are **out of your control**.

- I’m not able to control the vast majority of my lucid dreams (roughly 80%-100%).
- I’m not able to control most of my lucid dreams (roughly 60%-70%).
- I’m not able to control roughly 50% of my lucid dreams and able to control roughly 50% of my lucid dreams.
- I’m able to control most of my lucid dreams (roughly 60%-70%).
- I’m able to control the vast majority of my lucid dreams (roughly 80%-100%).

1. Try to estimate the **duration of lucidity** in the majority of your lucid dreams, based on the number of seconds. If the duration is inconstant, choose the longest duration which characterizes your lucid dreams.

- Once I realize that I’m dreaming - I wake up.
- Usually, the lucidity lasts for 1-10 seconds.
- Usually, the lucidity lasts for 11-60 seconds.
- Usually, the lucidity lasts for 2-5 minutes.
- Usually, the lucidity lasts for 6 minutes or more.

1. Try to estimate the **duration of lucidity** in the majority of your lucid dreams, based on the number of dream scenes. If the duration is inconstant, choose the longest duration which characterizes your lucid dreams.

- Once I realize that I’m dreaming - I wake up.
- Usually, I experience 1 lucid dream scene.
- Usually, I experience 2 lucid dream scenes.
- Usually, I experience 3 lucid dream scenes.
- Usually, I experience 4 lucid dream scenes or more.

**In order to answer the next 4 questions, try to** **estimate the percentage of lucid dreams in which you experience the event described in the question.**

1. Try to estimate in what percentage of your lucid dreams, you remember that the dream started out with a positive tone (note that this question refers to the beginning of the dream, or a moment before lucidity onset).

| 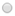 | 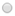 | 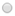 | 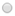 | 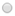 | 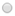 | 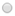 | 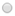 | 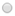 | 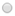 | 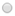 |
| --- | --- | --- | --- | --- | --- | --- | --- | --- | --- | --- |
| 100% | 90 | 80 | 70 | 60 | 50 | 40 | 30 | 20 | 10 | 0% |
| All lucid dreams start out as positive |  |  |  |  |  |  |  |  |  | No lucid dream starts out as positive |

1. Try to estimate in what percentage of your lucid dreams, you remember that the dream started out with a negative tone, or was a nightmare (note that this question refers to the beginning of the dream, or a moment before lucidity onset).

| 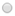 | | 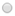 | | 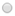 | | 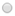 | | 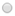 | | 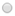 | 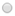 | 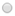 | | 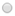 | | 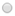 | | 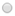 | |
| --- | --- | --- | --- | --- | --- | --- | --- | --- | --- | --- | --- | --- | --- | --- | --- | --- | --- | --- | --- |
| 100% | 90 | | 80 | | 70 | | 60 | | 50 | | 40 | | 30 | | 20 | | 10 | | 0% |
| All lucid dreams start out as negative | |  | |  | |  | |  | |  |  |  | |  | |  | | No lucid dream starts out as negative | |

1. Try to estimate in what percentage of your lucid dreams, you remember that the lucidity brought along with it a positive emotion, i.e., you felt good after becoming lucid (e.g., happy, relieved, excited, etc.).

| 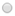 | | 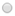 | | 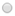 | | 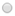 | | 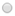 | | 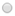 | 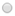 | | 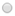 | | 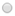 | | 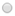 | | 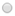 | |
| --- | --- | --- | --- | --- | --- | --- | --- | --- | --- | --- | --- | --- | --- | --- | --- | --- | --- | --- | --- | --- |
| 100% | 90 | | 80 | | 70 | | 60 | | 50 | | | 40 | | 30 | | 20 | | 10 | | 0% |
| I always feel good after becoming lucid | |  | |  | |  | |  | |  |  | |  | |  | |  | | I never feel good after becoming lucid | |

1. Try to estimate in what percentage of your lucid dreams, you remember that the lucidity brought along with it a negative emotion, i.e., you felt bad after becoming lucid (e.g., fearful, anxious, sad, etc.).

| 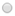 | | 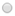 | | 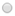 | | 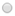 | | 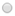 | | 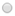 | 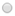 | | 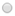 | | 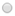 | | 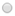 | | 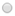 | |
| --- | --- | --- | --- | --- | --- | --- | --- | --- | --- | --- | --- | --- | --- | --- | --- | --- | --- | --- | --- | --- |
| 100% | 90 | | 80 | | 70 | | 60 | | 50 | | | 40 | | 30 | | 20 | | 10 | | 0% |
| I always have a bad feeling after becoming lucid | |  | |  | |  | |  | |  |  | |  | |  | |  | | I never have a bad feeling after becoming lucid | |

**Section C (Optional)**

This part of the questionnaire is designated only for those who have attempted to initiate lucid dreams at least once. If you replied “I have never attempted to initiate lucidity in a dream deliberately” in question 4, please skip this part of the questionnaire.

The next questions refer to the frequency with which you used, or are using, various techniques aimed to induce lucid dreams. Mark “X” in one slot in every row under the answer which is most accurate according to your experiences.

| *3 times a week or more during the last month* | *Once or twice a week during the last month* | *Up to 3 times during the last month* | *In the past, but not during the last month* | *never* |  |  |
| --- | --- | --- | --- | --- | --- | --- |
|  |  |  |  |  | I keep a dream diary and record my dreams in it when I wake up | 15 |
|  |  |  |  |  | I read about lucid dreams | 16 |
|  |  |  |  |  | I think of lucid dreams when I’m awake | 17 |
|  |  |  |  |  | I perform reality checks to increase the frequency of my lucid dreams (e.g., counting fingers, reading text, using electrical appliances, moving through walls) | 18 |
|  |  |  |  |  | I plan sleeping and waking times in a way that increases lucid dreams occurrences | 19 |
|  |  |  |  |  | I use advanced techniques to induce lucid dreams, such as MILD (Memory Induced Lucid Dreams) or WILD (Wake Induced Lucid Dreams) | 20 |
|  |  |  |  |  | Before I fall asleep, I think about lucid dreams and wish for them | 21 |

**Section S2**

**Daily Lucid Dreaming Diary**

*Subject ID: _____________ Date: _____________ Time: _____________*

1. Did you experience a lucid dream tonight?

- Yes

If you replied “yes” to this question, please proceed to the next questions.

באם לא, דלג על חלק זה בשאלון ועבור לחלק ג'.

- No

1. Was the lucidity **spontaneous or deliberately initiated**?

- Spontaneous
- Initiated

1. **How long** did the lucidity last?

- A few seconds to 1 minute
- 1 minute to 5 minutes
- 5 to 10 minutes
- More than 10 minutes

1. To what extent did you experience your lucid dream as **realistic and tangible**?

- Very low
- Low
- Moderate
- High
- Very high

1. To what degree did you experience **awareness** in your lucid dream (i.e., the understanding that this is a dream)?

- Very low
- Low
- Moderate
- High
- Very high

1. Did you **attempt** to control the events of the dream volitionally?

- Yes
- No

1. If you replied “yes” in the question 19, answer: to what extent did you **succeed** in controlling and manipulating the events of the dream volitionally?

- Very low
- Low
- Moderate
- High
- Very high

1. How did you **achieve lucidity** in the dream?

- I can’t remember
- I noticed some strangeness in the dream scene
- I performed a reality check
- WILD (Wake Induced Lucid Dreams)
- MILD (Memory Induced Lucid Dreams)
- Other: _________________

Comments:

______________________________________________________________________________________________________________________________________________________________________________________________________________________________________________________

| **Frequency** | (0) never | | | (1) only at childhood | | | | | (2) <1 a year | | | | | (3) 1-2 a year | (4) 3-11 a year | (5) 1-2 a month | (6) 3-4 a month | (7) ≥2 week | | | | | N/A | Skewness | | | | | Kurtosis |
| --- | --- | --- | --- | --- | --- | --- | --- | --- | --- | --- | --- | --- | --- | --- | --- | --- | --- | --- | --- | --- | --- | --- | --- | --- | --- | --- | --- | --- | --- |
| 1. Momentary | 21.39 | | | 9.09 | | | | | 20.86 | | | | | 15.51 | 20.86 | 9.09 | 3.21 | 0.00 | | | | | 0.00 | 0.05 | | | | | -1.05 |
| 2. Prolonged | 37.97 | | | 13.37 | | | | | 14.97 | | | | | 13.37 | 14.44 | 5.35 | 0.53 | 0.00 | | | | | 0.00 | 0.50 | | | | | -1.06 |
| 3. Spontaneous | 21.39 | | | 11.76 | | | | | 20.32 | | | | | 13.37 | 21.39 | 9.09 | 2.14 | 0.00 | | | | | 0.53 | 0.08 | | | | | -1.12 |
| 4. Attempt | 64.17 | | | 11.76 | | | | | 7.49 | | | | | 6.95 | 4.81 | 3.21 | 0.53 | 0.53 | | | | | 0.53 | 1.73 | | | | | 2.23 |
| 5. Success | 15.5 | | | 2.7 | | | | | 6.4 | | | | | 4.8 | 4.8 | 1.1 | 0.5 | 0 | | | | | 64.2 | 0.6 | | | | | -0.82 |
| **Intensity** | | 0 - uncertain / passive / not in control 80-100% of LD | | | | | | 1 - uncertain / passive / not in control 60-70% of LD | | | | | 2 - confident / active / in control 50% of LD | | 3 - confident / active / in control 60-70% of LD | | 4 - confident / active / in control 80-100% of LD | | | N/A | | | | Skewness | | Kurtosis | | | |
| 6. Confidence | | 21.93 | | | | | | 16.58 | | | | | 11.76 | | 12.83 | | 12.30 | | | 24.60 | | | | 0.29 | | -1.31 | | | |
| 7. Activity | | 25.13 | | | | | | 14.97 | | | | | 12.83 | | 12.83 | | 8.02 | | | 26.20 | | | | 0.41 | | -1.16 | | | |
| 8. Control | | 33.16 | | | | | | 18.18 | | | | | 10.70 | | 8.56 | | 2.14 | | | 27.27 | | | | 0.89 | | -0.29 | | | |
| **Length** | (0) waking up at the moment of lucidity | | | | | | (1) 1-10 seconds / one dream scene | | | | (2) 11-60 seconds / 2 dream scenes | | | | (3) 2-5 minutes / 3 dream scenes | | (4) ≥ 6 minutes / ≥ 4 dream scenes | | | | N/A | | | Skewness | | | Kurtosis | | |
| 9. Length - seconds | 12.30 | | | | | | 29.95 | | | | 17.65 | | | | 12.83 | | 1.60 | | | | 25.67 | | | 0.37 | | | -0.63 | | |
| 10.Length - scenes | 13.90 | | | | | | 40.11 | | | | 13.90 | | | | 5.35 | | 1.07 | | | | 25.67 | | | 0.82 | | | 0.80 | | |
| **Emotional valence** | | | 0-10% | | 10-20% | | | | | 20-30% | | | | 30-40% | 40-50% | 50-60% | 60-70% | 70-80% | 80-90% | | | | 90-100% | N/A | Skewness | | | | Kurtosis |
| 11. Positive beginning | | | 5.88 | | 4.28 | | | | | 9.63 | | | | 7.49 | 19.25 | 4.28 | 5.35 | 6.42 | 6.42 | | | | 4.28 | 26.74 | 0.21 | | | | -0.79 |
| 12. Negative beginning | | | 9.63 | | 6.95 | | | | | 6.95 | | | | 3.21 | 16.04 | 5.88 | 8.02 | 5.88 | 4.28 | | | | 4.28 | 28.88 | 0.10 | | | | -0.97 |
| 13. Positive ending | | | 5.88 | | 1.60 | | | | | 4.28 | | | | 4.28 | 8.02 | 5.88 | 8.02 | 10.16 | 10.16 | | | | 14.97 | 26.74 | -0.60 | | | | -0.72 |
| 14. Negative ending | | | 24.06 | | 9.63 | | | | | 12.83 | | | | 2.67 | 8.02 | 3.21 | 4.28 | 0.53 | 1.60 | | | | 2.67 | 30.48 | 1.12 | | | | 0.45 |
| **Techniques** | (0) never | | | | | (1) in the past, but not during the last month | | | | | | (2) ≤3 during the last month | | | (3) 1-2 a week during the last month | | (4) ≥3 a week during the last month | | | | | N/A | | Skewness | | | | Kurtosis | |
| 15. Dream diary | 23.53 | | | | | 9.63 | | | | | | 1.07 | | | 0.00 | | 0.00 | | | | | 65.78 | | 1.28 | | | | 0.73 | |
| 16. Reading | 18.72 | | | | | 14.97 | | | | | | 0.53 | | | 0.00 | | 0.00 | | | | | 65.78 | | 0.45 | | | | -1.12 | |
| 17. Thoughts at daytime | 10.16 | | | | | 16.04 | | | | | | 6.42 | | | 1.60 | | 0.00 | | | | | 65.78 | | 0.55 | | | | -0.14 | |
| 18. Reality checks | 22.99 | | | | | 9.63 | | | | | | 1.07 | | | 0.53 | | 0.00 | | | | | 65.78 | | 1.78 | | | | 3.74 | |
| 19. Planning sleep time | 30.48 | | | | | 3.74 | | | | | | 0.00 | | | 0.00 | | 0.00 | | | | | 65.78 | | 2.56 | | | | 4.72 | |
| 20. Advanced techniques | 28.88 | | | | | 4.28 | | | | | | 1.07 | | | 0.00 | | 0.00 | | | | | 65.78 | | 2.55 | | | | 6.07 | |
| 21. Thoughts at bedtime | 14.44 | | | | | 13.90 | | | | | | 2.67 | | | 3.21 | | 0.00 | | | | | 65.78 | | 1.06 | | | | 0.41 | |

**Supplementary Tables**

***Table S1.***

*Distribution of the FILD items (data presented in percentages)*

***Table S2.***

*Correlations, Means, and Standard Deviations of FILD items*

|  | 1 | 2 | 3 | 4 | 5 | 6 | 7 | 8 | 9 | 10 | 11 | 12 | 13 | 14 | 15 | 16 | 17 | 18 | 19 | 20 | 21 | M | SD |
| --- | --- | --- | --- | --- | --- | --- | --- | --- | --- | --- | --- | --- | --- | --- | --- | --- | --- | --- | --- | --- | --- | --- | --- |
| 1 | - |  |  |  |  |  |  |  |  |  |  |  |  |  |  |  |  |  |  |  |  | 2.45 | 1.75 |
| 2 | .60^***^ | - |  |  |  |  |  |  |  |  |  |  |  |  |  |  |  |  |  |  |  | 1.71 | 1.69 |
| 3 | .79^***^ | .69^***^ | - |  |  |  |  |  |  |  |  |  |  |  |  |  |  |  |  |  |  | 2.38 | 1.74 |
| 4 | .27^***^ | .32^***^ | .24^**^ | - |  |  |  |  |  |  |  |  |  |  |  |  |  |  |  |  |  | 0.90 | 1.51 |
| 5 | .54^***^ | .74^***^ | .49^***^ | .68^***^ | - |  |  |  |  |  |  |  |  |  |  |  |  |  |  |  |  | 1.61 | 1.70 |
| 6 | .20^*^ | .25^**^ | .28^**^ | .07 | .25 | - |  |  |  |  |  |  |  |  |  |  |  |  |  |  |  | 1.70 | 1.46 |
| 7 | .03 | .28^**^ | .09 | .21^*^ | .08 | .33^***^ | - |  |  |  |  |  |  |  |  |  |  |  |  |  |  | 1.51 | 1.39 |
| 8 | .10 | .35^***^ | .13 | .24^**^ | .28^*^ | .32^***^ | .56^***^ | - |  |  |  |  |  |  |  |  |  |  |  |  |  | 1.01 | 1.16 |
| 9 | .10 | .45^***^ | .21^*^ | .20^*^ | .25 | .19^*^ | .22^**^ | .43^***^ | - |  |  |  |  |  |  |  |  |  |  |  |  | 1.48 | 1.03 |
| 10 | .10 | .38^***^ | .25^**^ | .13 | .20 | .16 | .28^**^ | .48^***^ | .54^***^ | - |  |  |  |  |  |  |  |  |  |  |  | 1.19 | 0.87 |
| 11 | -.08 | .04 | -.07 | .04 | .03 | .02 | .15 | .16 | .02 | .05 | - |  |  |  |  |  |  |  |  |  |  | 49.80 | 25.22 |
| 12 | .12 | -.10 | .08 | .04 | -.02 | .04 | -.11 | -.11 | -.02 | -.05 | -.70^***^ | - |  |  |  |  |  |  |  |  |  | 46.79 | 26.71 |
| 13 | .13 | .18^*^ | .18^*^ | .07 | .09 | .36^***^ | .15 | .28^**^ | .26^**^ | .27^**^ | .17^*^ | -.06 | - |  |  |  |  |  |  |  |  | 63.68 | 28.45 |
| 14 | -.19^*^ | -.14 | -.21^*^ | .06 | .01 | -.28^**^ | -.01 | -.10 | -.17 | -.24^**^ | -.08 | .32^***^ | -.60^***^ | - |  |  |  |  |  |  |  | 28.77 | 26.26 |
| 15 | .00 | -.03 | -.08 | .19 | .06 | -.15 | .09 | -.10 | -.10 | -.20 | -.02 | -.23 | -.06 | .02 | - |  |  |  |  |  |  | 0.34 | 0.54 |
| 16 | -.13 | -.08 | -.10 | .12 | -.09 | .03 | -.01 | -.03 | -.17 | .07 | .10 | .17 | .15 | .02 | .20 | - |  |  |  |  |  | 0.47 | 0.53 |
| 17 | .36^**^ | .48^***^ | .40^**^ | .47^***^ | .45^**^ | .10 | -.02 | .13 | .18 | .04 | .08 | .06 | .20 | -.03 | .44^***^ | .34^**^ | - |  |  |  |  | 0.98 | 0.83 |
| 18 | .31^*^ | .31^*^ | .24 | .31^*^ | .32^*^ | .16 | .15 | .23 | .31^*^ | .28^*^ | -.08 | .07 | .19 | -.18 | .25^*^ | .34^**^ | .44^***^ | - |  |  |  | 0.39 | 0.63 |
| 19 | .01 | .09 | .06 | .14 | .12 | .00 | .16 | .13 | .04 | .01 | .01 | .13 | .02 | .00 | .15 | .07 | .31^*^ | .34^**^ | - |  |  | 0.11 | 0.31 |
| 20 | -.03 | -.08 | .00 | .09 | -.06 | -.12 | .18 | .24 | -.01 | .08 | .25 | -.05 | .17 | -.11 | .18 | .15 | .30^*^ | .23 | .40^**^ | - |  | 0.19 | 0.47 |
| 21 | .15 | .27^*^ | .20 | .47^***^ | .30^*^ | .09 | .09 | .21 | .18 | .03 | .24 | -.02 | .25 | -.09 | .11 | .25 | .41^**^ | .40^**^ | .39^**^ | .29^*^ | - | 0.84 | 0.93 |

Items: 1 - Momentary frequency, 2 - Prolonged frequency, 3 - Spontaneous frequency, 4 - Attempt frequency, 5 - Success frequency, 6 - Confidence, 7 - Activity, 8 - Control, 9 - Length by seconds, 10 - Length by scenes, 11 - Positive beginning, 12 - Negative beginning, 13 - Positive ending, 14 - Negative ending, 15 - Dream diary, 16 - Reading, 17 - Thoughts - daytime, 18 - Reality checks, 19 - Planning sleep time, 20 - Advanced, 21 - Thoughts - sleep.

* p < .05. **p < .01. *** p < .001.

1. Item 5 was applicable only to those who answered in an affirmative manner to item 4. [↑](#footnote-ref-1)
